# Factors associated with non-invasive ventilation response on the first day of therapy in patients with hypercapnic respiratory failure

**DOI:** 10.1186/cc9570

**Published:** 2011-03-11

**Authors:** G Gürsel, M Aydoğdu, S Taşyürek, G Gülbaş, S Özkaya, S Nazik, A Demir

**Affiliations:** 1 Gazi University Medical Faculty, Ankara, Turkey; 2 İnönü University Medical Faculty, Pulmonary Diseases Department, Malatya, Turkey; 3 Rize University Medical Faculty, Pulmonary Diseases Department, Rize, Turkey

## Introduction

Non-invasive ventilation (NIV) decreases the need for mechanical ventilation in the early period of acute hypercapnic respiratory failure and factors for success have been studied well. On the other hand, little is known about what kind of factors influence the NIV response in the subacute period. This study aimed to determine the factors influencing PaCO_2 _reduction below 50 mmHg in the first 24 hours of therapy.

## Methods

In this retrospective study we investigated the differences in NIV strategies and patient characteristics between the responsive group (PaCO_2 _levels drop below 50 mmHg in first 24 hours) (group 2) and the nonresponsive group (group 1).

## Results

In 34% of the patients, PaCO_2 _reduced to below 50 mmHg in first 24 hours. There were no significant differences between the length of NIV application time and ICU stay, intubations and mortality rates, across the groups. Despite a significantly higher level of pressure support usage in group 1 than in group 2, PaCO_2 _did not reduce below 50 mmHg in group 1 within the first 24 hours. While 91% of the responsive group had received nocturnal NIV therapy, only 74% of the nonresponsive group had received NIV therapy all night long (*P *= 0.036). The home ventilation usage rate was significantly higher in the nonresponsive group than the responsive group.

## Conclusions

Results of this study showed that, although nocturnal application of NIV in the ICU is associated with a faster drop rate in PaCO_2 _levels, the higher pressure support requirement and prior home ventilation usage are predictors for late and poorer response to NIV.

